# The potential of positive deviance approach for the sustainable control of neglected tropical diseases

**DOI:** 10.1186/s41182-016-0023-8

**Published:** 2016-07-13

**Authors:** Ken Ing Cherng Ong, Hitomi Araki, Shigeyuki Kano, Masamine Jimba

**Affiliations:** Department of Community and Global Health, Graduate School of Medicine, The University of Tokyo, Tokyo, Japan; Department of Tropical Medicine and Malaria, Research Institute, National Center for Global Health and Medicine, Tokyo, Japan

**Keywords:** Neglected tropical disease, Positive deviance

## Abstract

Neglected tropical diseases (NTDs) have gained much attention in recent years due to the support from various agencies. However, the main approach to combat NTDs has been to cure rather than to prevent. As many NTD infections are closely linked with human behaviors such as hygienic practices and tradition, behavior change is also very crucial to prevent relapse or reinfection. Therefore, we would like to suggest a potential new approach—the positive deviance approach—to tackle NTDs by focusing on the preventive phase. What makes this approach unique is that the solution comes from the affected population themselves and not from the expert outsiders. Preventive chemotherapy that relies on outside aid has serious sustainability issues as reinfection is also high after the aid program has ended. Learning from the success story in Vietnam on preventing childhood malnutrition, the positive deviance approach could end the spread of NTDs once and for all by making full use of the available local solutions.

## Main text

As the chapter on the Millennium Development Goals (MDGs) has ended, the United Nations set another target that would presumably steer the world in a better direction: the Sustainable Development Goals (SDGs). The target concerning health and diseases that scourge the humankind in SDGs has been reworded from “*to combat HIV/AIDS, malaria and other diseases’* to *‘to end the epidemics of AIDS, tuberculosis, malaria and neglected tropical diseases(NTDs)*” [1]. Therefore, instead of continuing the fight, we should strive to make these diseases a history once and for all.

By looking at the transmission routes, NTDs are very closely linked to human behaviors and hygienic practices. For example, soil-transmitted helminth infections, echinococcosis, and trachoma can be avoided by adopting good personal hygiene practices such as hand-washing and not going out barefoot [2]. Transmission of schistosomiasis, cysticercosis, and foodborne trematode infections can be interrupted by maintaining good sanitation practices such as avoiding open defecation [2]. Infection of lymphatic filariasis, human African trypanosomiasis, Chagas disease, dengue, and onchocerciasis can be discontinued by keeping the surrounding clean and sustaining a vector-free environment [2]. Finally, cysticercosis and foodborne trematode infections are associated with deeply entrenched cultural practices such as consumption of raw pork or fish. Therefore, the affected population needs a difficult but not impossible behavior change [3].

The WHO has outlined five strategies to prevent, control, eliminate, and eradicate neglected tropical diseases. These strategies are preventive chemotherapy, intensified disease management, vector and intermediate host control, veterinary public health at the human-animal interface, and provision of safe water, sanitation and hygiene [4]. As NTDs have garnered much-needed attention in recent years, many global partnerships have been formed and several pharmaceutical companies have pledged free drugs to combat NTDs worldwide [4]. Preventive chemotherapy has been shown to be effective in reducing the prevalence and morbidity of NTDs but reinfection with the parasite is also very common among endemic populations [5–7].

The current trend in global health research implementation is to carry out various interventions and programs to improve the health status of the population at risk based solely on what worked previously, oftentimes in a different context [8]. This trend stems from the fact that many stakeholders are determined to see changes in terms of something tangible, in this case, significant statistics [8]. More often than not, interventions were treatment-oriented and solely aimed at alleviating the current complication that were plaguing the target population. For instance, although preventive chemotherapy by means of mass drug administration (MDA) could bring down the prevalence, the reinfection rate is also high [5–7]. This intervention through MDA alone brings the issue of sustainability into question. Therefore, to achieve a sustainable control or elimination and eradication of NTDs, tools that complement the current arsenal against NTDs are needed.

Compared to other behavior change communication (BCC) approaches, positive deviance is a concept or an approach where the focus is not on what did not work but instead on what actually worked [9]. Unlike other BCC approaches where the outside health experts tend to work together with the community to come up with a new solution for a problem, the positive deviance approach assumes that the solution already exists in the community. The role of the outside health expert is to work together with the community to promulgate this unique solution to the whole community [9, 10]. Furthermore, traditional approaches, such as television advertisement, radio messages, and posters are often based on the needs to contain a certain disease and the community might find it difficult to sustain the behavior. In addition, when a community is constantly exposed to these messages, a saturation point might be reached and further efforts to change the community’s behavior would be in vain and the impact achieved is low [10, 11].

Typically, the usual approach to solve a local health problem is to look for outside solutions and the focus is on what did not work [8, 9]. Local solutions and local wisdom are normally brushed aside [8]. For example, in Vietnam, this approach was employed against childhood malnutrition problem in the early 1990s. Rather than focusing on the high prevalence of malnutrition, the researchers approached the small group of well-nourished children. To their surprise, they found that the caregivers fed their children with small shrimps and other food which was deemed a taboo for the children but was freely available from the rice field. In addition, the caregivers also washed their children’s hands whenever they found their hands were dirty, and increased the daily feeding frequency. Within two years, most of the under-nourished children recovered and their status was sustained [9].

The positive deviance approach may be a viable health communication approach when a problem is not entirely technical but requires behavior change. It is also effective when other solutions have not worked and where positive deviants are believed to exist, and sponsorship and local leadership commitment are available to confront the issue [9, 10]. Basically, the positive deviance process begins with the community defining the problems and elucidating the desired outcome. Next, the community identifies the usual behavior and also discovers unusual but advantageous behavior through inquiry. Then, the community, together with the PD facilitators, designs and develops learning initiatives to disseminate the PD solutions*.* The PD approach is distinctive in a sense that the solutions to the problems are very local and unique to the particular community and is not a one-size-fit-all solution. For example, based on the success in Vietnam in tackling malnutrition, the solution was to feed the children with crabs, shrimps, and potato greens that are freely available in the village. This solution is unique and may not be applied to other geographical settings [9–12].

In the case of NTDs, the strategy is to identify individuals who are not affected by the disease of interest but have uncommon yet advantageous behaviors. These individuals are living in the same endemic area and have access to the same resources as those who are affected, but somehow, they are able to evade the disease. The positive deviance approach has been used in the case of Chagas disease control program in Ecuador [12]. The conventional strategies in Chagas disease control in Ecuador such as insecticide fumigation and community education were found to be effective only in the short term and not sustainable in preventing the reinfestation of the triatomines. Therefore, this study zoomed in on the households that were able to maintain a triatomine-free environment and identified common practices in these households. The triatomine-free households, or the positive deviants, maintain practices such as sweeping their house with plants that are believed to have insect-repelling properties, keeping their domestic animals away from their houses, and fumigating their houses [12].

The positive deviance approach has the potential to complement and synergize current tools such as MDA to fight against NTDs as the solutions come from the affected people themselves and are based on what are available. Instead of focusing only on what is lacking and what should be done, it is high time we looked at what is available and what could be done.

## Abbreviations

All explained in the main text.
